# Towards Reliable Transient Stability Prediction of Power Systems: A CNN-Based Deep Ensemble Model with Optimized Class-Specific Thresholds

**DOI:** 10.3390/s26154767

**Published:** 2026-07-27

**Authors:** Zhen Chen, Qiyu Liu, Hangtian Xiong, Chang Liu, Yankai Xing

**Affiliations:** 1 College of Nuclear Technology and Automation Engineering, Chengdu University of Technology, Chengdu 610059, China; chenzhen5840@cdut.edu.cn (Z.C.); 2025050583@stu.cdut.edu.cn (Q.L.); 2025050578@stu.cdut.edu.cn (H.X.); 2 Sichuan New Electric Power System Research Institute, Chengdu 610041, China

**Keywords:** transient stability prediction, phasor measurement units, CNN-based deep ensemble, class-specific thresholds, confidence estimation

## Abstract

The wide deployment of phasor measurement units has enabled data-driven transient stability prediction (TSP) of power systems. However, ensuring the reliability of TSP results is still a significant challenge that limits the practical application of data-driven methods. To this end, a convolutional neural network (CNN)-based deep ensemble model with optimized class-specific thresholds is proposed to achieve reliable TSP. Specifically, a CNN is utilized as the backbone predictor, where the time-series variables from multiple generators are transformed into image-like inputs, and a CNN-based deep ensemble model is developed to provide accurate confidence estimation for TSP. Subsequently, considering the asymmetric importance of different classes in TSP, a confidence-based class-specific thresholds rule is adopted, and a multi-objective optimization model for determining the class-specific thresholds is formulated. In this optimization model, the reliability requirement of TSP is imposed as a constraint, requiring that true unstable rate (TUR) equal to 100%, with the objectives of minimizing the rejection rate and maximizing the true stable rate (TSR). The Pareto front of the class-specific thresholds can be obtained by solving the optimization model. Test results on two benchmark power systems show that the proposed method achieves a TUR of 100% and a TSR of at least 99% with approximately 10% of the samples rejected, demonstrating its effectiveness and scalability.

## 1. Introduction

Transient stability is the ability of a power system to retain synchronism subject to large disturbances, and it is widely recognized as a key prerequisite for ensuring the secure operation of power systems [1]. Once transient instability occurs, it may trigger generator tripping, or even cascading events that culminate in a power system blackout. With renewable energy resources and power electronic devices integrated on a large scale, operating conditions become more variable, and post-fault system dynamics exhibit stronger nonlinearity and greater complexity, posing more significant challenges to transient stability analysis [2]. In this context, it is imperative to develop fast and reliable transient stability analysis methods to ensure stable system operation in modern power systems.

Currently, transient stability assessment in actual power systems still relies predominantly on the time-domain simulation method based on component-level physical models [3]. By modeling system components in detail, the method can capture the system dynamics under fault disturbances with high accuracy, and thus offer advantages in terms of reliability and model applicability [4]. However, due to the increasingly complex grid dynamics and highly variable operating modes in modern power systems, time-domain simulation often takes excessive time and cannot meet the requirements of online transient stability analysis [5].

With the wide deployment of phasor measurement units (PMU) and rapid development of artificial intelligence, data-driven transient stability prediction (TSP) of power systems using pre-fault or post-fault data has become a frontier research direction [6,7,8]. Typically, TSP is formulated as a binary classification problem, where the operating condition after a fault is labeled as either stable or unstable. Various learning models, including extreme learning machines [9,10], convolutional neural networks (CNN) [11,12,13,14], long short-term memory networks [15,16], transformer [17], evolving symbolic model [18] and other models, have been applied to TSP with promising performance. However, since data-driven methods are inherently based on statistical learning, it is difficult to completely eliminate prediction errors. This issue is particularly critical in high-risk applications such as TSP, where even a small number of misclassifications may lead to severe consequences, which limits the practical application of data-driven methods for TSP.

To improve the reliability of TSP, one practical approach is to provide a quantitative confidence measure for each sample together with the stability prediction result. By introducing a decision threshold, low-confidence samples can be rejected to mitigate decision risk. The subsequent treatment of these rejected samples depends on the application scenario: in pre-fault scenarios, they can be verified via time-domain simulation, whereas in post-fault scenarios, the observation window can be extended to gather more dynamic information for a more reliable decision [9,19]. However, when implementing this approach, two key challenges arise. The first is how to accurately estimate the confidence of TSP outputs, which should faithfully reflect model uncertainty. The second is how to reasonably determine the decision threshold under the performance requirements of TSP.

For confidence estimation, the most common practice is to use the maximum softmax probability (MSP) of a neural network as the confidence metric [11,12,13]. In [20,21], the bayesian neural network (BNN) is utilized to output predictive probabilities for TSP. Moreover, the confidence neural network (ConfidNN) using the true class probability (TCP) as the confidence metric is developed in [22]. However, these methods, which are constructed with a single model, generally suffer from overconfidence, where incorrectly predicted samples are often assigned excessively high confidence, and cannot provide accurate confidence estimates [23,24,25,26]. To address this, some studies have explored ensemble architectures for TSP confidence estimation. For instance, bootstrap ensemble methods are used in [9,19], and a multi-kernel CNN ensemble model with MSP is introduced in [27]. Despite these efforts, further improvement in confidence estimation accuracy is still essential for enhancing overall TSP performance.

As for decision threshold determination, most existing methods rely on empirical settings, making it difficult to guarantee optimal model performance [9,13,25]. In [10], a multi-objective confidence threshold optimization model is proposed by jointly considering metrics such as accuracy and decision earliness. However, the asymmetric importance of different stability classes is not adequately accounted for, where instability detection must be prioritized over stability detection [25,28]. As a result, the optimized threshold may fail to strictly satisfy reliability criteria such as zero missed detection of unstable samples.

To address the above challenges, a CNN-based deep ensemble model with optimized class-specific thresholds is proposed for reliable TSP. With the post-fault dynamic trajectories as raw data, the time-series variables from multiple generators are transformed into image-like inputs [12,27]. Therefore, the CNN is utilized as the backbone predictor, and the resulting deep ensemble model provides accurate confidence estimation for TSP. Subsequently, considering the asymmetric importance of different classes in TSP, a confidence-based class-specific thresholds rule is adopted, and a multi-objective optimization model for determining the class-specific thresholds is formulated. In this optimization model, the reliability requirement of TSP is imposed as a constraint, requiring that true unstable rate (TUR) equal to 100%, with the objectives of minimizing the rejection rate (RR) and maximizing the true stable rate (TSR).

The main contributions of this work are summarized as follows:

(1) A CNN-based deep ensemble model is developed for confidence estimation in TSP with PMU data. By leveraging the model diversity introduced by random initialization under the same training data and fixed network architecture, the proposed model provides more accurate confidence estimation for TSP.
(2) A class-specific thresholds optimization model is formulated to determine the decision thresholds. In this model, the TSP reliability requirement is taken as the constraint, thereby yielding the Pareto front of class-specific thresholds that strictly satisfy the TSP reliability requirement.
(3) A reliable TSP method combining the CNN-based deep ensemble model and the class-specific thresholds optimization strategy is proposed. Benefiting from more accurate confidence estimation, the proposed method can provide a superior Pareto front and thus improve overall evaluation efficiency.

The remainder of this paper is organized as follows. Section 2 presents the proposed methodology in detail. Section 3 describes the overall procedure of the proposed reliable TSP scheme. Comprehensive case studies are presented in Section 4. Section 5 discusses practical deployment issues and the computational cost. The conclusions are given in Section 6.

## 2. Proposed Methodology

This section presents the proposed methodology, including the CNN-based deep ensemble model and the class-specific thresholds optimization strategy. The proposed model is developed to provide more accurate confidence estimation for TSP. Based on the confidence estimation results, the class-specific thresholds optimization strategy is further presented to meet the reliability requirement of TSP.

### 2.1. CNN-Based Deep Ensemble Model

#### 2.1.1. Outline of CNN

As a powerful tool for image processing, the CNN has been widely used in image classification tasks [29]. In this paper, the post-fault dynamic trajectories from PMUs are treated as raw data, and the time-series variables from multiple generators are transformed into image-like inputs, making CNN a natural choice as the backbone predictor for TSP.

In a CNN, the input is first processed by a convolution operation and then passes through a rectified linear unit (ReLU) for nonlinear activation. The output of the convolutional layer *C* is given by:

(1) $$C=f\left(W_{1}⊗X+b_{1}\right)=\mathrm{ReLU}\left(W_{1}⊗X+b_{1}\right)$$

where *X* denotes the input, *W*_1_ and *b*_1_ denote the convolution kernel and bias, respectively, and ⊗ denotes the convolution operation.

A max-pooling layer is then employed for down-sampling, and the output of the pooling layer *P* is given by:

(2) $$P=\mathrm{MaxPool}\left(C\right)$$

where MaxPool(·) denotes the maximum pooling operation.

The pooled features are then flattened into a one-dimensional vector and fed into fully connected layers. Finally, the class probabilities are obtained via the softmax layer:

(3) $$p_{j}=\frac{\exp\left(z_{j}\right)}{\sum_{j=1}^{k}\exp\left(z_{j}\right)},j=1,\ldots ,k$$

where *z*_j_ denotes the logit of the *j*-th class, *p*_j_ denotes the corresponding predicted probability, and *k* is the number of classes (i.e., *k* = 2 corresponding to the stable and unstable classes in this paper).

**Figure 1:**
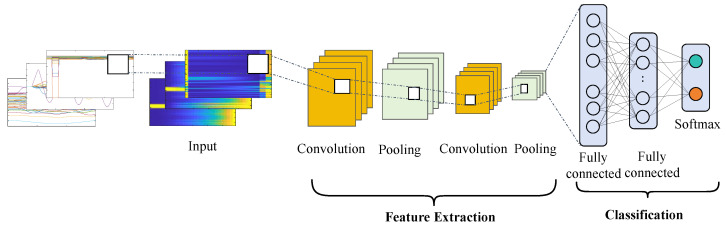
Architecture of the CNN for TSP.

The architecture of the CNN used in this paper is shown in Figure 1 [12], consisting of two main modules: a feature extraction module and a classification module. The feature extraction module comprises two cascaded stages, each consisting of a convolution layer followed by a pooling layer. The classification module maps the extracted features through two fully connected layers. Finally, a softmax layer outputs the predicted probabilities of the stable and unstable classes.

#### 2.1.2. Proposed CNN-Based Deep Ensemble Model

To obtain more accurate confidence estimation of TSP outputs, the deep ensemble framework, which is a simple yet powerful method for predictive uncertainty estimation [23,24], is employed. Owing to the diversity introduced by random parameter initialization, deep ensembles have been shown to yield superior uncertainty estimation compared with traditional bootstrap ensemble methods [30].

**Figure 2:**
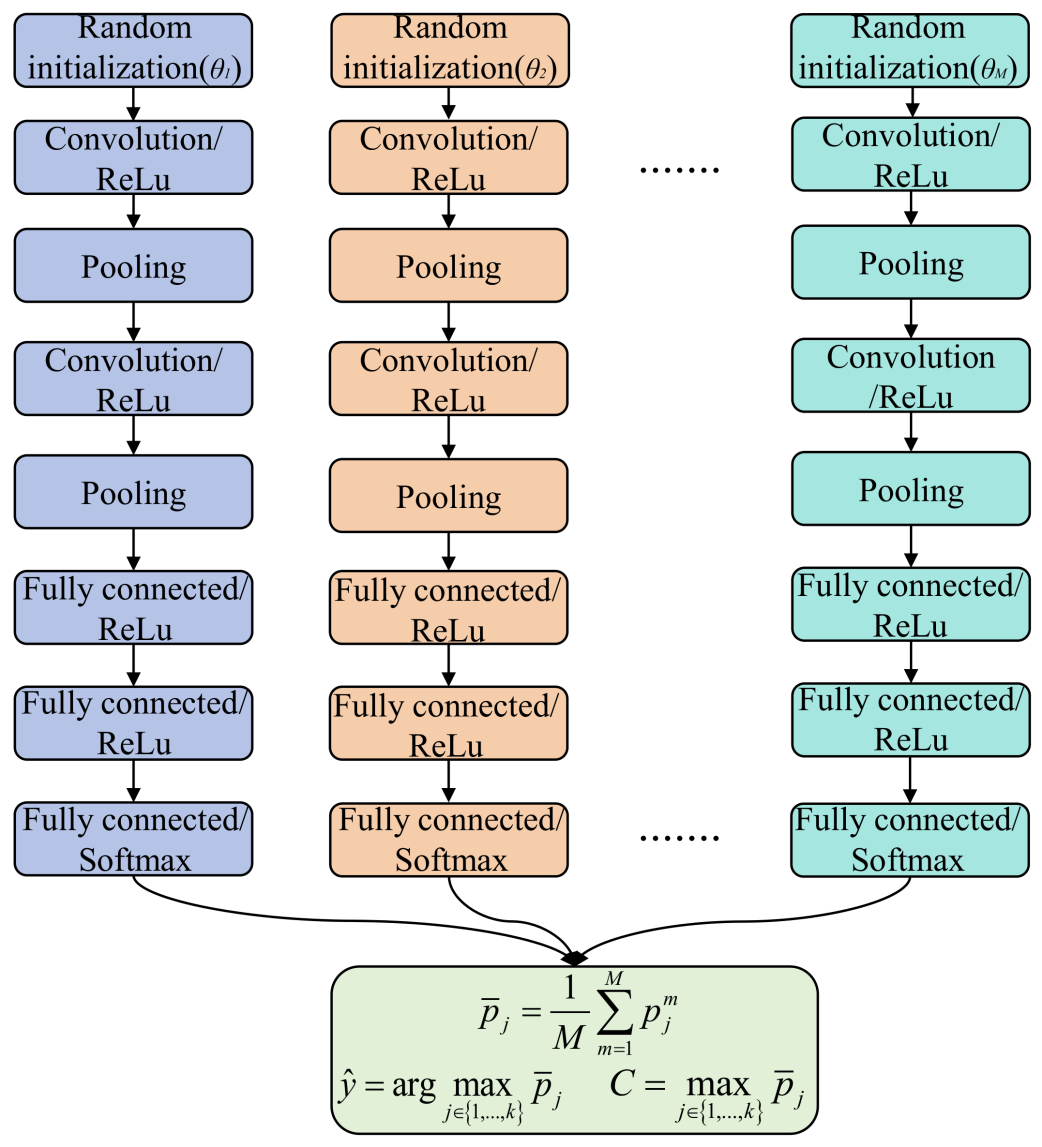
Architecture of the CNN-based deep ensemble model.

In this work, the previously described CNN is used as the base predictor within the deep ensemble. Multiple CNN subnetworks, each sharing the same architecture and training data, are constructed and trained independently, differing only in their random initializations. The final predicted probabilities are obtained by averaging the outputs of all subnetworks, which leads to more accurate confidence estimates. The overall architecture of the proposed CNN-based deep ensemble is illustrated in Figure 2.

In the CNN-based deep ensemble architecture, the input is processed in parallel by *M* CNN subnetworks with different random parameter initializations. In each CNN subnetwork, the input is processed through convolution and pooling operations for feature extraction. The extracted features are then mapped to a logit vector by the fully connected layer. After that, the logit vector is converted into class probabilities through a softmax layer. The softmax operation of the m-th subnetwork is shown as:

(4) $$p_{j}^{m}=\frac{\exp\left(z_{j}^{m}\right)}{\sum_{j=1}^{k}\exp\left(z_{j}^{m}\right)},m=1,\ldots ,M$$

where $p_{j}^{m}$ denotes the predicted probability of the *j*-th class as produced by the *m*-th subnetwork, and $z_{j}^{m}$ denotes the corresponding logit.

The final ensemble prediction probability is then obtained by averaging the output probabilities from all *M* CNN subnetworks, which is shown as:

(5) $${\bar{p}}_{j}=\frac{1}{M}\sum_{m=1}^{M}p_{j}^{m}$$

where ${\bar{p}}_{j}$ is the ensemble prediction probability outputs of *j*-th class.

The MSP of the CNN-based deep ensemble is deemed as the confidence metric and the predicted class $\bar{y}$ and the associated prediction confidence *C* are determined as follows:

(6) $$\left\{\begin{array}{l} \bar{y}=\arg\underset{j\in \left\{1,\ldots ,k\right\}}{\max}{\bar{p}}_{j}\\ C=\underset{j\in \left\{1,\ldots ,k\right\}}{\max}{\bar{p}}_{j} \end{array}\right.$$

### 2.2. Class-Specific Thresholds Optimization Strategy

After the confidence of the prediction result is obtained, an empirically selected threshold is commonly applied for decision-making. Samples with confidence lower than this threshold are rejected. Prediction results are provided only for samples whose confidence exceeds the threshold, so that the accuracy of the retained samples can be ensured [9]. However, such a single threshold rejection strategy is based on the assumption that all classes are equally important, and the asymmetric importance of different classes in practical applications is therefore not considered.

**Figure 3:**
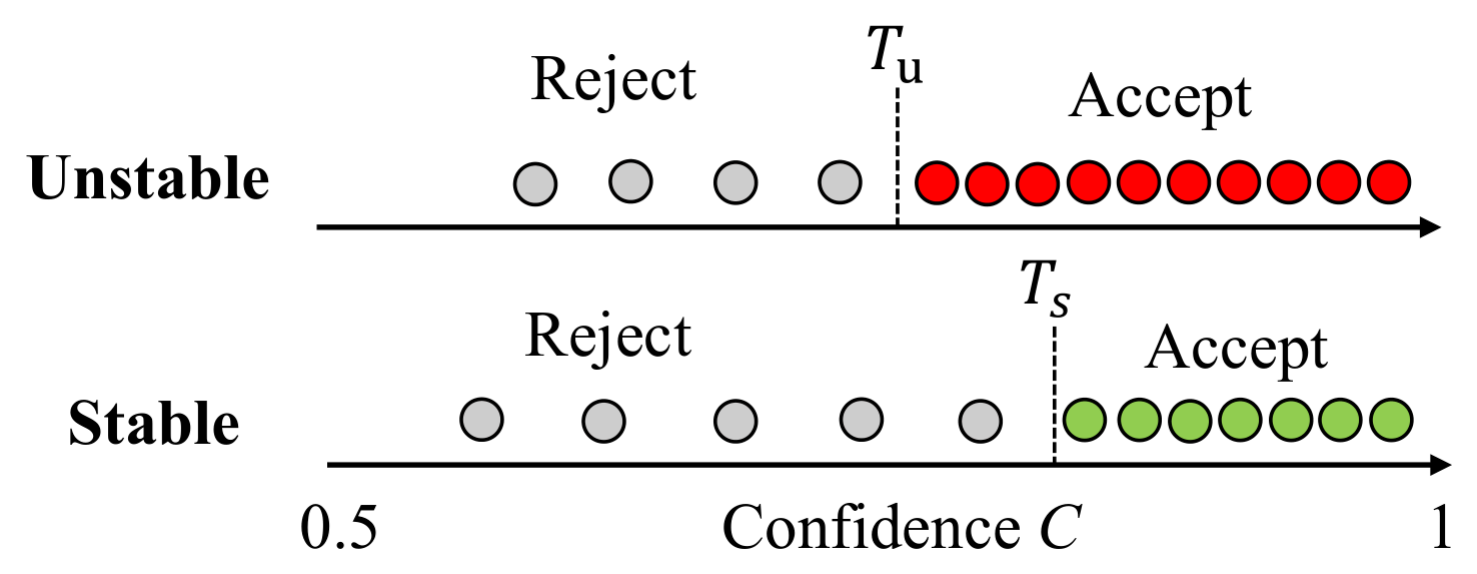
Illustration of confidence-based class-specific thresholds rule.

For TSP, the importance of unstable samples is significantly higher than that of stable samples. If an unstable sample is misclassified as stable, dispatchers cannot perceive the instability risk in time to take control measures, which can easily trigger large-scale blackouts. Conversely, if a stable sample is misclassified as unstable, certain economic losses or unnecessary control actions may be caused, but stable operation of the power system is generally not endangered [25]. Therefore, based on the confidence of the prediction result, a class-specific thresholds rule is adopted [28]. As illustrated in Figure 3, different confidence thresholds are assigned to the unstable and stable classes, denoted by *T_u_* and *T_s_*, respectively. When *T_u_* < *T_s_*, a stricter decision criterion is applied to the stable class, which reflects the asymmetric importance of different classes. In this way, the proposed class-specific thresholds can effectively reduce the misclassification of unstable samples as stable, thereby improving the identification accuracy of the unstable class.

Based on the class-specific thresholds rule, the decision of TSP can be expressed as:

(7) $$y=\left\{\begin{array}{l} 1\;if\;{\bar{p}}_{1}\leq {\bar{p}}_{2}\;\mathrm{and}\;C>T_{u}\\ -1\;if\;{\bar{p}}_{1}>{\bar{p}}_{2}\;\mathrm{and}\;C>T_{s}\\ r\;\mathrm{otherwise} \end{array}\right.$$

where ${\bar{p}}_{1}$ and ${\bar{p}}_{2}$ denote the predicted probabilities of the stable and unstable classes, respectively. In addition, *y* represents the final decision result, with 1 and −1 corresponding to the labels of the unstable and stable classes, respectively. *r* indicates that the sample is rejected by the prediction model.

A sample is assigned to the unstable or stable class only when both the predicted label and its associated confidence satisfy the corresponding threshold requirements. Otherwise, the sample is rejected and verified by further analysis.

To quantitatively evaluate the classification performance under different class-specific thresholds, the TSR, TUR, and RR are defined as:

(8) $$\mathrm{TSR}=\frac{TS}{TS+FU}$$

(9) $$\mathrm{TUR}=\frac{TU}{TU+FS}$$

(10) $$\mathrm{RR}=\frac{N_{r}}{N}$$

where *TS* represents the number of stable samples correctly predicted, *FU* denotes the number of stable samples misclassified as unstable, *TU* refers to the number of unstable samples correctly predicted, and *FS* indicates the number of unstable samples misclassified as stable. Additionally, *N_r_* is the number of rejected samples, and *N* is the total number of samples.

When selecting class-specific thresholds, it is necessary to meet the reliability requirements of the TSP decision results:

(1) All unstable samples are correctly classified, satisfying the reliability requirement of TSP, that is, TUR = 100% [31,32].
(2) Under the condition of satisfying requirement (1), TSR should be maximized while RR is minimized. Specifically, low confidence samples are assigned to a rejection set. Given the significant computational burden of these simulations, minimizing RR is essential to maintain overall evaluation efficiency.

Based on the above analysis, a multi-objective optimization model for the class-specific thresholds can be formulated as follows:

(11) $$\begin{array}{l} \min\;\mathrm{RR}\\ \max\;\mathrm{TSR}\\ s.t.\;\mathrm{TUR}=100\%\\ \;0.5\leq T_{u}\leq 1\\ \;0.5\leq T_{s}\leq 1 \end{array}$$

The non-dominated sorting genetic algorithm II (NSGA-II) [33] is used to solve the above multi-objective optimization problem and obtain the Pareto front of class-specific thresholds. The constraint TUR = 100% is handled using a penalty function so that the selected thresholds satisfy the prescribed TSP reliability requirement.

## 3. Reliable TSP Scheme

**Figure 4:**
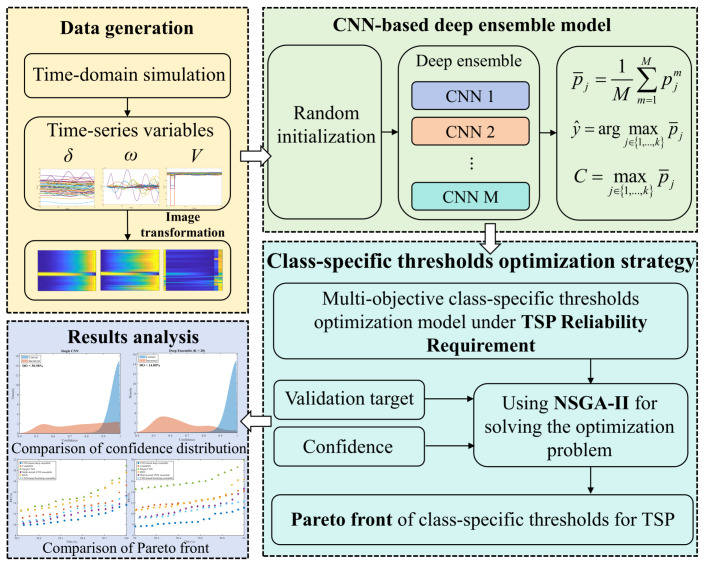
Flowchart of the proposed reliable TSP scheme.

Figure 4 illustrates the overall flowchart of the proposed reliable TSP scheme, which specifically includes the following steps:

(1) **Data generation**: By considering diverse operating conditions and fault disturbances, a large set of samples is generated via time-domain simulations. For each fault scenario, three types of time-series variables from generators, namely rotor angle *δ*, rotor speed *ω*, and voltage magnitude *V*, are taken as the raw inputs, which can be obtained by PMUs during online application. To enable faster early response and better capturing of dynamic characteristics in TSP, the “fault-on+2” time window is adopted [34]. This is achieved by extending the fault duration to include one adjacent sample both before fault occurrence and after fault clearance. All sample labels are determined using the stability index *η* [35], which is defined as:

(12) $$\eta =\frac{360^{\circ }-\left|\Delta \delta_{\max}\right|}{360^{\circ }+\left|\Delta \delta_{\max}\right|}$$

where Δ*δ*_max_ denotes the maximum rotor angle deviation between any pair of generators in the system.

The sign of *η* determines the stability label: *η* > 0 indicates a stable case labeled as −1, whereas *η* < 0 indicates an unstable case labeled as 1. In essence, the criterion assesses post-fault synchronism by comparing the value of Δ*δ*_max_ with a predefined critical threshold during the simulation period. The “fault-on+2” trajectories of {*δ*, *ω*, *V*} are first normalized using z-score standardization and are transformed into image-like inputs. The resulting labeled database is then split into three subsets, namely the training set, validation set, and test set.

(2) **CNN-based deep ensemble model**: The image-like inputs obtained in the previous step are fed into the CNN-based deep ensemble model, and the model is trained using the training set. The proposed model is formed by training *M* CNN subnetworks independently with different random initializations and fixed network architecture, using the cross-entropy loss function and Adam optimizer [36]. For a given sample, the output class probabilities from all CNN subnetworks are averaged to obtain the confidence estimates and the predicted label. The accurately estimated confidence values can be used for the subsequent class-specific thresholds optimization strategy.

(3) **Class-specific thresholds optimization strategy**: Based on the class-specific thresholds rule, a class-specific thresholds optimization model is formulated subject to TUR = 100% as a constraint, with the objectives of maximizing TSR and minimizing RR. Using the validation set, NSGA-II is employed to solve the optimization problem taking into account the validation targets and their corresponding confidence estimates, and the Pareto front of class-specific thresholds is produced to satisfy the TSP reliability requirement while maintaining computational efficiency.

(4) **Results analysis**: the test set is used to evaluate the performance of the proposed scheme, including the confidence estimation results and the effectiveness of the class-specific thresholds optimization strategy.

## 4. Case Study

To evaluate the effectiveness and scalability of the proposed method, case studies are conducted on two benchmark power systems, namely, the IEEE 68-bus system and NPCC 140-bus system. The entire workflow, including time-domain simulations, data processing, and model implementation, is performed in MATLAB 2024b. Specifically, the Power System Toolbox 3.0 [37] is utilized for time-domain simulation. All experiments are executed on a computer equipped with an Intel Core i7-14650HX CPU (2.2 GHz) (Intel Corporation, Santa Clara, CA, USA), 32 GB RAM, and an NVIDIA RTX-5070 Ti Laptop GPU (NVIDIA Corporation, Santa Clara, CA, USA). In addition, the CNN hyperparameters listed in Table 1 are determined according to the physical characteristics of the data, while the remaining hyperparameters are empirically set to ensure sufficient feature learning.

### 4.1. Tests on the IEEE 68-Bus System

#### 4.1.1. Simulation Settings and Data Generation

**Figure 5:**
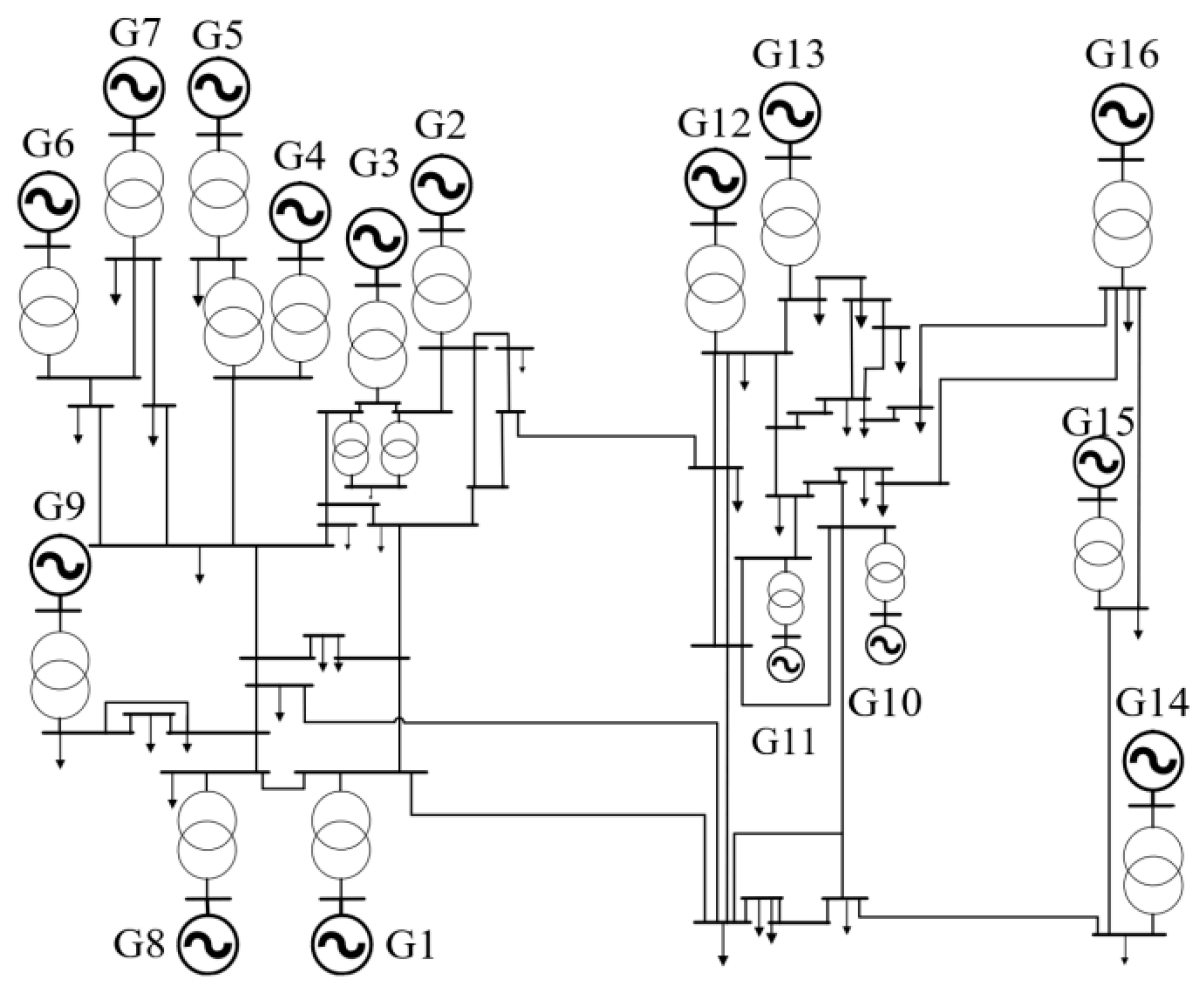
Single-line diagram of the IEEE 68-bus system.

The proposed method is tested on the IEEE 68-bus system in this section. The single-line diagram of the IEEE 68-bus system is illustrated in Figure 5 [38]. Data generation starts from the 100% base load-flow condition. The load demand at each bus is randomly perturbed within 80–120% of its base value to create different loading levels. Subsequently, one transmission line is randomly selected as the faulted line, and a three-phase short-circuit fault is applied. The fault is initiated at t = 0.1 s, and the fault clearing time is varied from 0.2 s to 0.3 s to reflect the impact of different protection schemes. For each scenario, the total simulation time is set to 5 s for stability label determination. The time-series variables of {*δ*, *ω*, *V*} are assumed to be obtained by PMU at a reporting rate of 120 Hz for each case.

Following the simulation set-up, a total of 8000 samples are generated, including 5980 stable samples and 2020 unstable samples. To facilitate model training and performance evaluation, the dataset is randomly divided into training, validation, and test sets in a ratio of 3:1:1.

#### 4.1.2. Confidence Estimation Results

**Figure 6:**
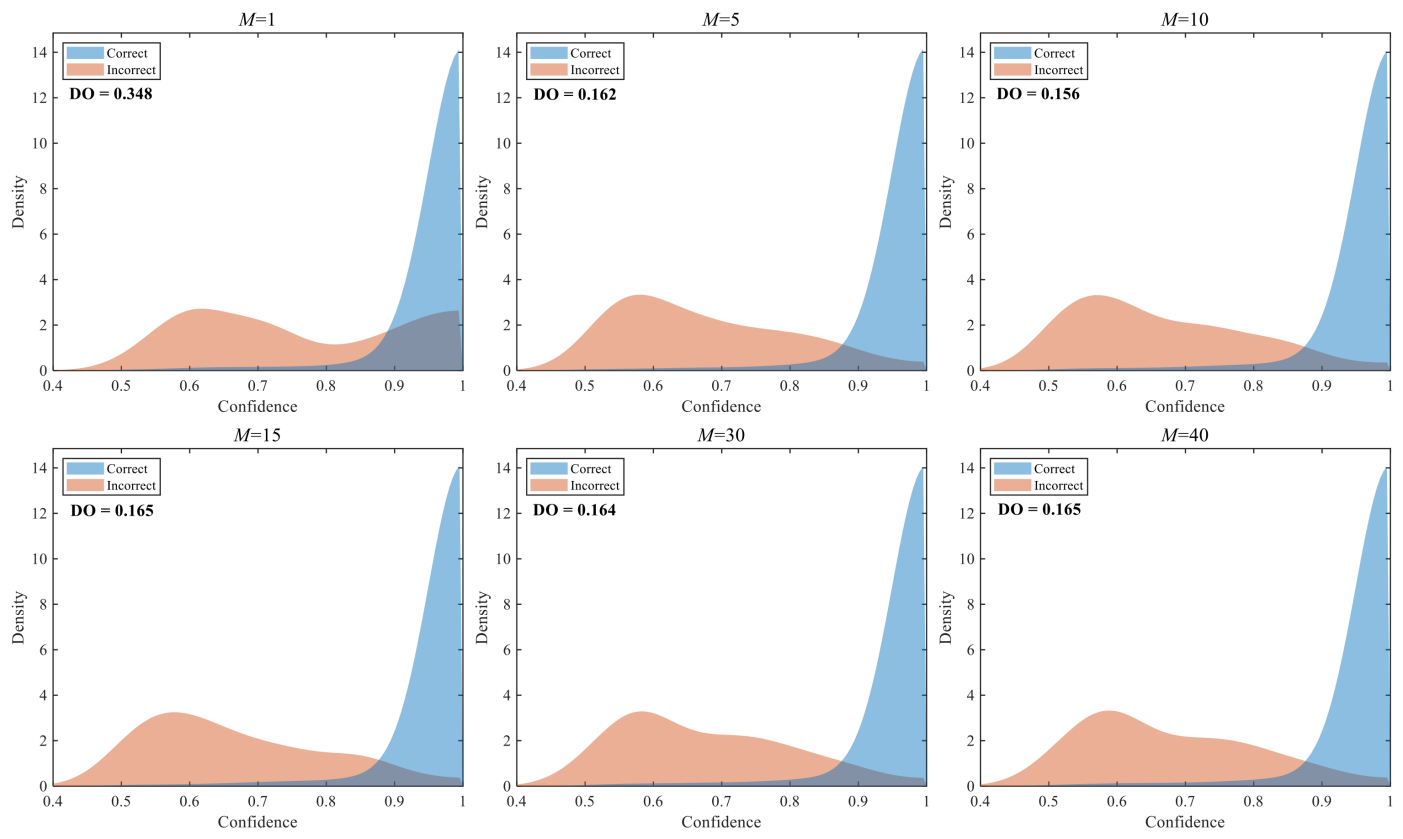
Confidence distributions with different ensemble sizes.

To show the confidence estimation results of the CNN-based deep ensemble for TSP, Figure 6 illustrates the confidence density profiles of correctly and incorrectly predicted samples, which are estimated using kernel density estimation. Furthermore, the area of confidence distribution overlap (DO) [39] of correct and incorrect predictions is adopted as a metric to quantitatively evaluate the separability of confidence distributions.

Figure 6 shows the confidence distributions of the CNN-based deep ensemble model on the validation set with different ensemble sizes, i.e., *M* = 1, 5, 10, 15, 30, and 40. The horizontal axis denotes the confidence of the prediction result, and the vertical axis denotes the sample density. The blue and orange curves correspond to correctly classified and incorrectly classified samples, respectively. For the single CNN model (*M* = 1), a pronounced overconfidence phenomenon can be observed: many incorrectly classified samples are assigned high confidence, resulting in a large DO with correctly classified samples, which indicates limited discriminative ability of the confidence estimation. As the ensemble size increases, the confidence distributions of correctly classified and incorrectly classified samples become more separable, and the DO decreases. This suggests that the proposed model effectively alleviates overconfidence by reducing the proportion of high-confidence incorrect predictions, thereby improving the discriminative ability of confidence estimation.

**Figure 7:**
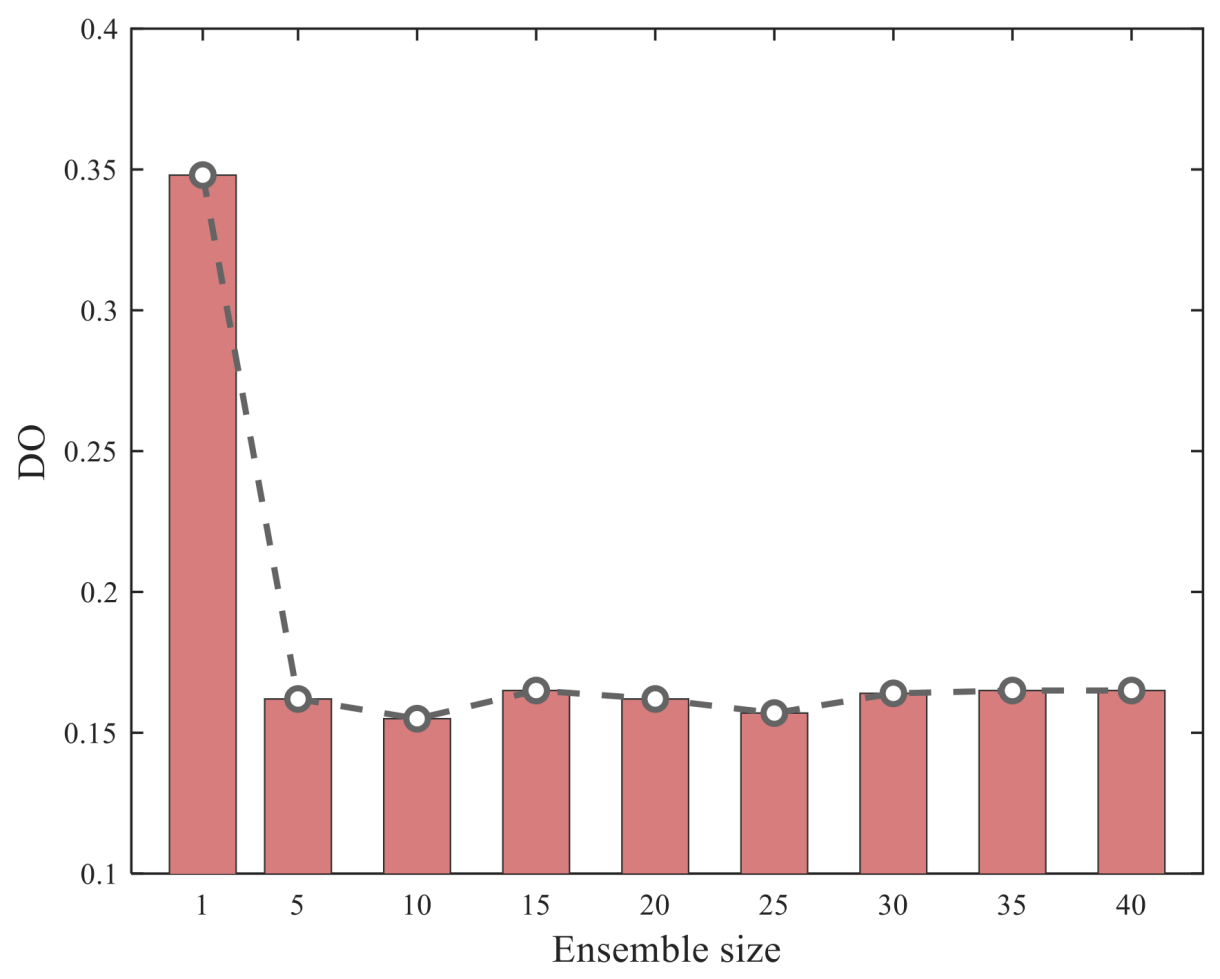
DO with different ensemble sizes on the IEEE 68-bus system.

The ensemble size *M* serves as a key factor determining the confidence estimation performance of the CNN-based deep ensemble. As illustrated in Figure 7, the DO metric exhibits a sharp initial decline with increasing ensemble size. Specifically, the DO value drops significantly from 0.348 at *M* = 1 to 0.155 at *M* = 10. Beyond *M* = 10, DO slightly increases and fluctuates within a small range. Therefore, according to the minimum DO, *M* = 10 is selected for all subsequent experiments.

To further evaluate the confidence estimation performance of the proposed model, comparisons are conducted with five representative models, including the single CNN [12], ConfidNN [22], multi-kernel CNN ensemble [27], BNN using MC dropout [40], and CNN-based bootstrap ensemble [9]. The single CNN adopts the architecture described in Table 1. The detailed configurations of the other four benchmark models are described as follows:

(1) **ConfidNN**: A multi-layer perceptron with four fully connected layers of 400 neurons each and a sigmoid output layer is trained on the extracted CNN features to estimate the TCP.
(2) **Multi-kernel CNN ensemble**: Three CNN subnetworks with kernel sizes of 3 × 3, 5 × 5 and 7 × 7 in the convolutional layer are constructed, while the remaining architecture is kept identical, and the output probabilities are averaged.
(3) **BNN**: MC dropout is applied to the same CNN architecture and remains active during inference. The final predictive probability is obtained by averaging 40 stochastic forward passes.
(4) **CNN-based bootstrap ensemble**: *M* bootstrap subsets are generated by sampling with replacement from the training data, each the same size as the training set. *M* CNNs are then trained independently, and the output probabilities are averaged.

The corresponding DO results are listed in Table 2. The results show that the proposed model achieves the lowest DO, indicating that the CNN-based deep ensemble model provides clearer separation between correctly classified and incorrectly classified samples in TSP.

#### 4.1.3. Class-Specific Thresholds Effect Analysis

**Figure 8:**
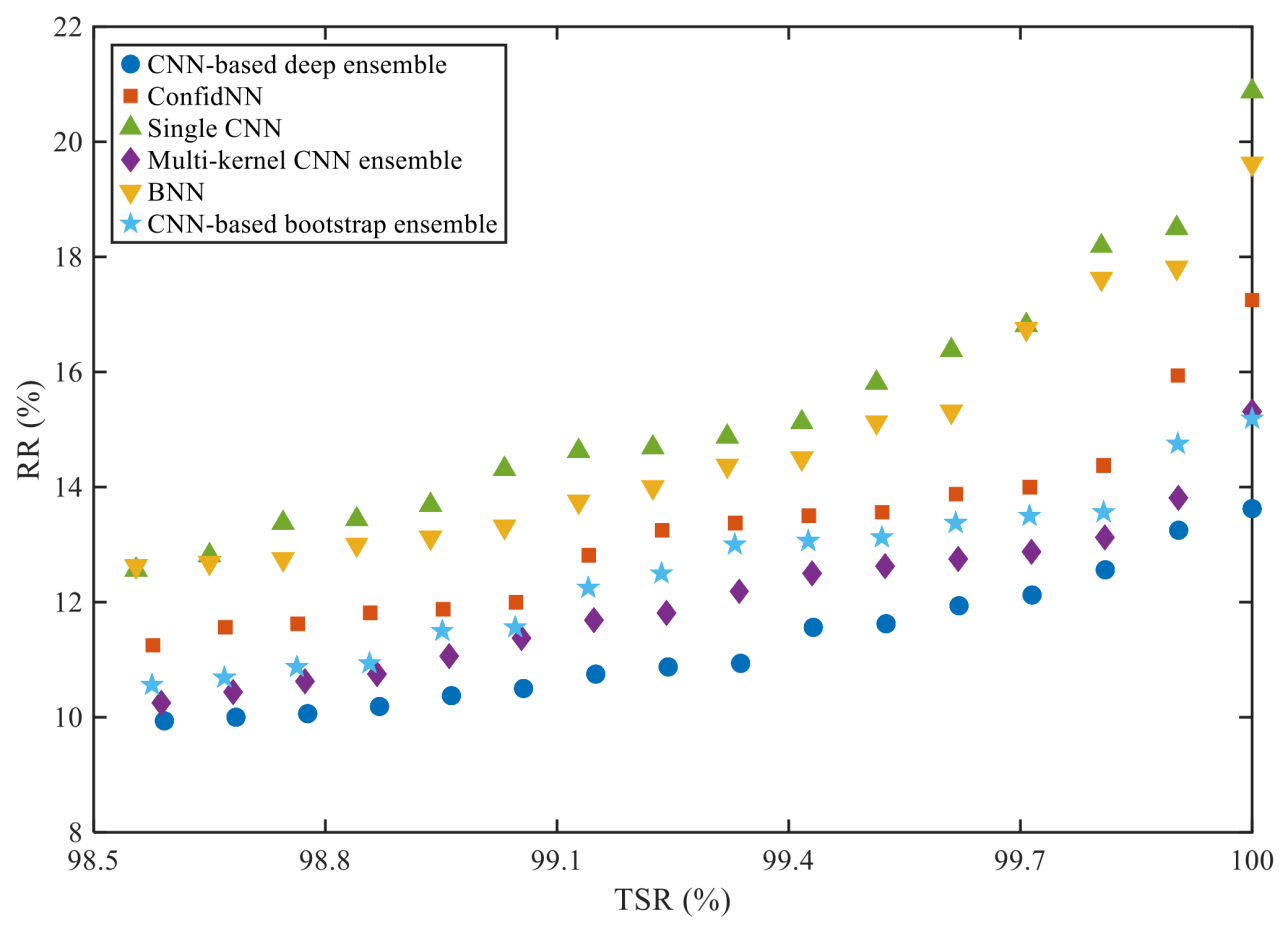
Pareto fronts of class-specific thresholds on the IEEE 68-bus system.

In this section, the class-specific threshold optimization strategy is applied using the validation set, based on the confidence estimation results obtained by the above four methods. The resulting Pareto fronts are illustrated in Figure 8.

To quantitatively compare the obtained Pareto fronts, Table 3 reports the RR values at TSR levels of approximately 99% and 100%, together with the hypervolume (HV) [41] of each Pareto front. For the HV calculation, the objectives were transformed into *f*_1_ = 100 -TSR and *f*_2_ = RR, and the reference point was set to (1.5,22).

As shown in Figure 8, the Pareto front obtained by the proposed method consistently lies below those of the competing methods. Table 3 further shows that the proposed method achieves RR values of 10.50% and 13.62% at TSR levels of approximately 99% and 100%, respectively, which are lower than those of all competing methods. It also achieves the highest HV of 16.08, indicating a better overall trade-off between TSR and RR.

The visual and quantitative results are consistent and collectively demonstrate that the proposed model provides more accurate confidence estimates, enables a superior Pareto front that achieves lower RR at comparable TSR levels and a higher HV.

Accordingly, five typical class-specific threshold combinations with TUR = 100% and TSR ≥ 99% are selected from the Pareto front of the proposed method in Figure 8, and Table 4 summarizes the corresponding performance on the validation and test sets.

These results indicate that the two decision thresholds play different roles. Specifically, raising *T_u_* reduces the risk of misclassifying stable samples as unstable, thereby mainly improving TSR, whereas adjusting *T_s_* ensures that unstable samples are not missed, thus mainly determining TUR. Moreover, the selected class-specific threshold combination achieves TUR = 100% on both the validation and test sets, satisfying the reliability requirement of TSP.

As shown in case 1, when *T_s_* = 0.969, TUR = 100% on the validation and test sets, indicating that no unstable sample is misclassified as stable and thus satisfying the reliability requirement of TSP. When *T_u_* = 0.698, the TSR on the validation and test sets are 99.06% and 99.34%, respectively, and the corresponding RR are 10.50% and 11%. On the test set, this RR of 11% means that about 11% of the samples are rejected and require further verification to determine their stability status, while the remaining 89% can be rapidly and reliably classified using the proposed method.

In case 5, to achieve TUR = 100% and TSR = 100% on both the validation and test sets, the selected class-specific threshold setting is *T_u_* = 0.895 and *T_s_* = 0.969. The RR on the test set is 13.12%.

### 4.2. Tests on the NPCC 140-Bus System

#### 4.2.1. Data Generation

**Figure 9:**
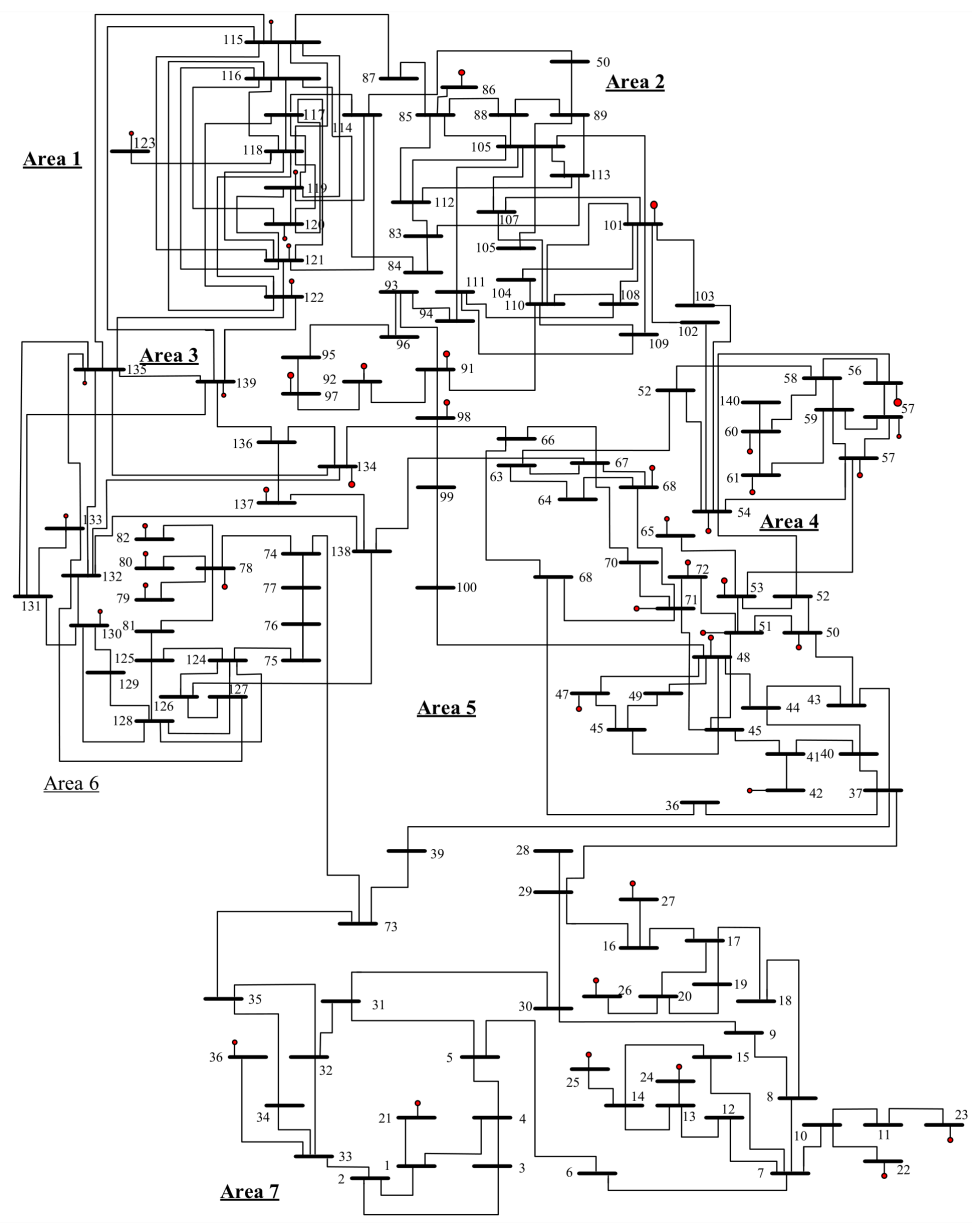
Single-line diagram of the NPCC 140-bus system.

The proposed method is further tested on the NPCC 140-bus system to evaluate its scalability. The single-line diagram of the system is illustrated in Figure 9 [42]. To ensure consistency in the experimental setup, the simulation set-up strategy described in Section 4.1.1 is adopted. Under the same load randomness ranges and fault configuration criteria, a total of 15,000 samples are generated, comprising 10,606 stable samples and 4394 unstable samples. The dataset is randomly divided into training, validation, and test sets in a ratio of 3:1:1.

#### 4.2.2. Confidence Estimation Results

**Figure 10:**
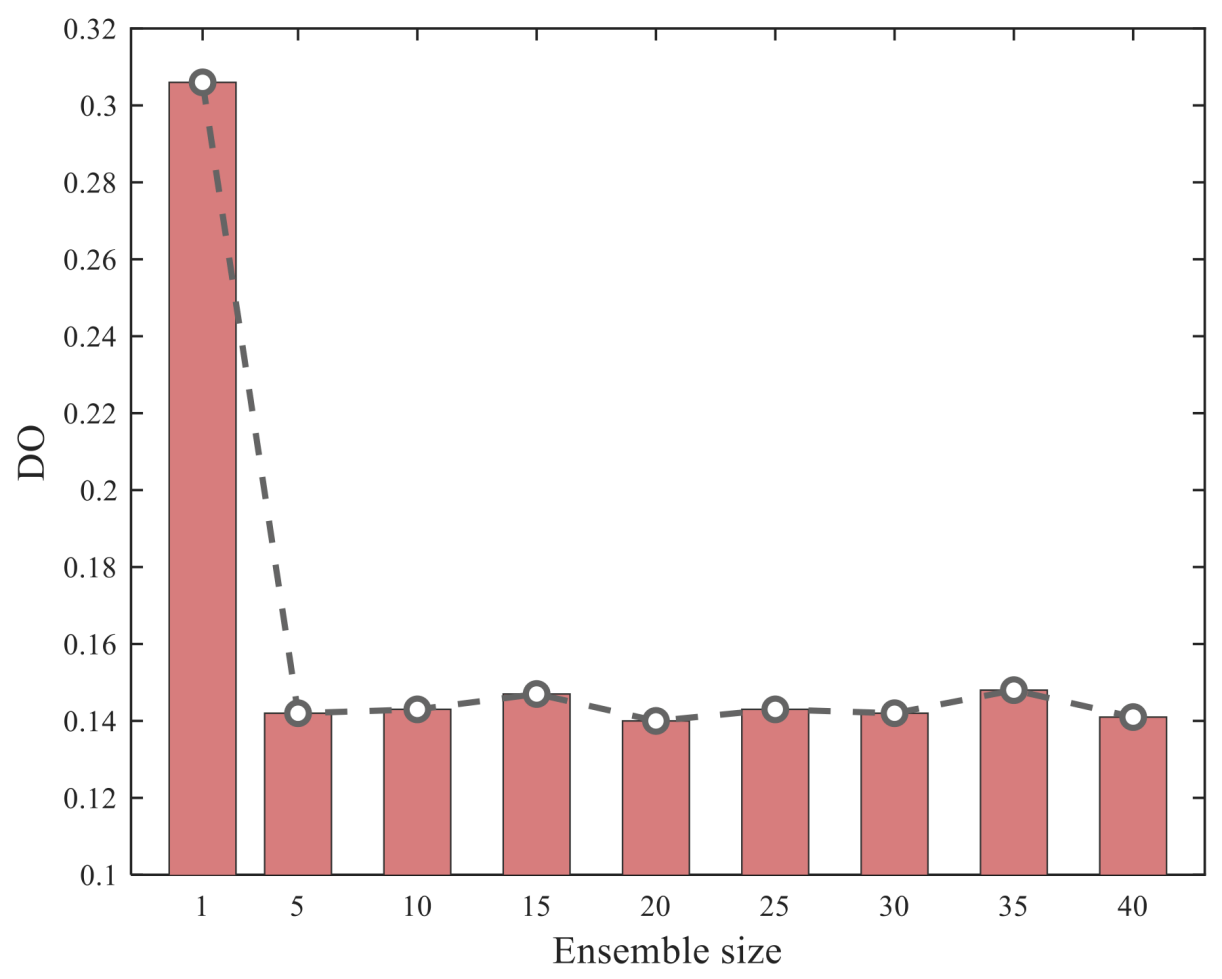
DO with different ensemble sizes on the NPCC 140-bus system.

Figure 10 illustrates the relationship between the DO metric and the ensemble size for the NPCC 140-bus system. As the ensemble size increases, the DO metric drops sharply at first, and then fluctuates within a narrow range when the size is further enlarged. Based on the minimum DO, *M* = 20 is selected for the subsequent experiments.

The DO results of the six models on the NPCC 140-bus system using the validation set are summarized in Table 5. Among the five compared models, the CNN-based deep ensemble model still achieves the lowest DO, indicating that its advantage in confidence estimation is preserved on the larger power system.

#### 4.2.3. Class-Specific Thresholds Effect Analysis

**Figure 11:**
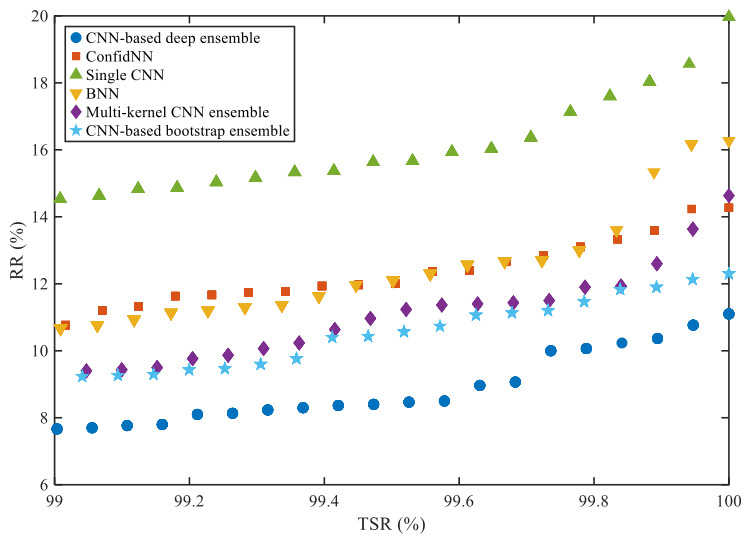
Pareto fronts of class-specific thresholds on the NPCC 140-bus system.

For the NPCC 140-bus system, the class-specific threshold optimization strategy is further applied to the confidence estimates obtained by the six models using the validation set. The Pareto fronts and quantitative comparison results are presented in Figure 11 and Table 6, respectively. For the HV calculation, the reference point was set to (1,22).

Figure 11 and Table 6 show that the proposed method produces a Pareto front below those of the competing methods, with the lowest RR values of 7.70% and 11.00% at TSR levels of approximately 99% and 100%, respectively, and the highest HV of 13.03. Together, these results confirm that the proposed model provides more accurate confidence estimates, enabling a superior Pareto front.

Based on the Pareto front obtained by the proposed method, five typical class-specific thresholds combinations with TUR = 100% and TSR ≥ 99% are selected from the Pareto front of the proposed method in Figure 11. Table 7 summarizes the corresponding performance on the validation and test sets.

For these selected thresholds, TUR = 100% on both the validation and test sets, indicating that no unstable sample is misclassified as stable and thus satisfying the reliability requirement of TSP.

As shown in case 1, when *T_s_* = 0.995, TUR = 100% on both the validation and test sets. When *T_u_* = 0.574, the TSR on the validation and test sets is 99.06% and 99.25%, with the corresponding RR being 7.70% and 9.10%, respectively. In case 5, to achieve TUR = 100% and TSR = 100% on both the validation and test sets, the selected class-specific thresholds setting is *T_u_* = 0.899 and *T_s_* = 0.995. The RR on the test set is 12.43%.

These results on the NPCC 140-bus system demonstrate the scalability of the proposed method to larger power systems.

## 5. Discussion

### 5.1. M Value Selection and Computational Cost Analysis

In this paper, the *M* value is treated as a hyperparameter and selected separately for each system by minimizing the validation DO from a predefined candidate range. It is very hard to give a clear physical justification for the relationship between *M* and the size/complexity of the power system, since the system dynamics and data distribution vary significantly across different systems, making it impractical to derive a general analytical expression. Instead, the same validation-based selection procedure can be applied independently to each test system in practice.

For the IEEE 68-bus system, the minimum validation DO is obtained at *M* = 10, whereas for the NPCC 140-bus system, it is obtained at *M* = 20. The computational costs of the single CNN and the ensemble model under sequential execution are reported in Table 8. For the IEEE 68-bus system with *M* = 10, the total training time is 13.02 min, and the inference latency per sample is 0.43 ms. For the NPCC 140-bus system with *M* = 20, the corresponding figures are 75.60 min and 0.96 ms, respectively.

Since all CNN subnetworks share the same architecture, differ only in random initialization, and are independent, the training and inference times scale roughly linearly with *M* under sequential execution. However, training is performed offline, so a moderate increase in training time does not impose a significant burden and does not affect practical deployment. The single-sample inference latency remains below 1 ms for both test systems, supporting the feasibility of the proposed method for online TSP applications. Moreover, the independence of the subnetworks enables efficient parallel execution, under which the overall time may theoretically approach that of a single CNN.

It should be noted that when *M* increases from 1 to 5, the DO decreases substantially from 0.348 to 0.162 on the IEEE 68-bus system, and from 0.288 to 0.142 on the NPCC 140-bus system. Beyond *M* = 5, the reduction in DO becomes marginal. Thus, *M* = 5 offers a practical trade-off when computational resources are constrained.

### 5.2. Practical Deployment

**Figure 12:**
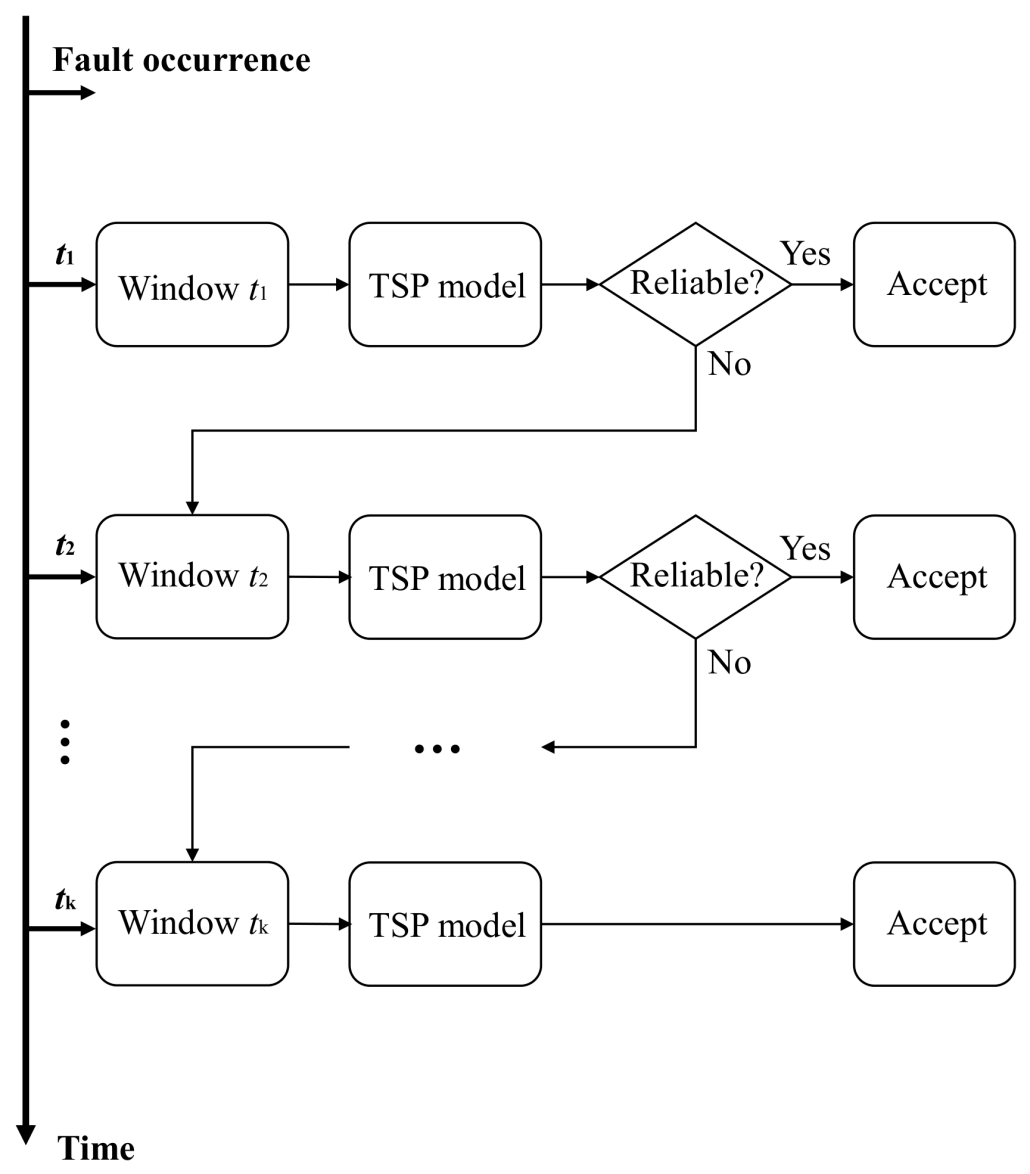
Practical deployment of the proposed reliable TSP scheme.

In practical deployment, dynamic responses are collected in real time by PMUs and directly fed into the model for online stability assessment. In post-fault scenarios, the system has already been perturbed, and the decision window is extremely tight. This is precisely why the “fault-on+2” time window is adopted as the default input in this work. However, such a short observation window inevitably leads to a fraction of samples for which the model cannot produce sufficiently reliable predictions. As shown in our results, achieving TUR = 100% and TSR ≥ 99% requires approximately 10% of samples to be rejected. For these uncertain samples, one viable strategy is to extend the trajectory observation length to gather more dynamic information, thereby improving the model’s confidence and discriminative ability, which aligns with the hierarchical rapid prediction framework in [15,19], whose detailed procedure is illustrated in Figure 12.

However, extending the observation window also reduces the time available for protection and emergency control. Therefore, a specific time constraint should be established according to the response requirements of the actual power system. If a reliable prediction is obtained within this constraint, it can be used for subsequent protection and control decision-making. If the model still cannot provide a reliable prediction when the time constraint is reached, the system should be conservatively treated as unstable, and the corresponding protection or emergency control measures should be taken to ensure system stability.

## 6. Conclusions

A reliable TSP method is proposed in this paper based on a CNN-based deep ensemble model with optimized class-specific thresholds. Results on the IEEE 68-bus and NPCC 140-bus systems validate the effectiveness and scalability of the proposed method. The main conclusions are summarized as follows:

(1) Compared with other models, the CNN-based deep ensemble model provides a clearer separation in confidence between correctly classified and incorrectly classified samples, thereby improving the accuracy of confidence estimation and mitigating the overconfidence problem.
(2) The proposed class-specific thresholds optimization strategy yields a Pareto front with respect to TSR and RR, and every threshold combination on this front satisfies the TSP reliability requirement, i.e., TUR is equal to 100%, thus supporting reliable TSP decision-making.
(3) By combining the proposed model with a class-specific thresholds optimization strategy, the resulting Pareto front yields a significantly lower RR than those obtained by other methods at comparable TSR levels, indicating that the proposed method provides a superior Pareto front and thus improves overall evaluation efficiency.

Future research will focus on two key issues. The first is how to construct an adaptive decision-making strategy that automatically selects the observation window according to power system operating conditions, thereby achieving a better balance between prediction reliability and decision speed. The second is how to further improve the deep ensemble model by exploring diversity enhancement approaches beyond random initialization, with the aim of improving confidence estimation accuracy and mitigating model overconfidence.

## Figures and Tables

**Table 1:** Hyperparameters of the CNN.

Layer	Architecture
Input	Input: Generator × Time × Variable
Convolution 1	3 × 3 kernel, 32 filters, stride 1, ReLU
Pooling 1	2 × 2 max-pooling, stride 2
Convolution 2	3 × 3 kernel, 64 filters, stride 1, ReLU
Pooling 2	2 × 2 max-pooling, stride 2
Flatten	Flatten Layer
Fully Connected 1	128 neurons, ReLU, Dropout (rate = 0.3)
Fully Connected 2	64 neurons, ReLU, Dropout (rate = 0.2)
Output	2 neurons, softmax

**Table 2:** DO comparison on the IEEE 68-bus system.

Method	DO
Single CNN	0.348
ConfidNN	0.242
Multi-kernel CNN ensemble	0.233
BNN	0.270
CNN-based bootstrap ensemble	0.234
CNN-based deep ensemble	**0.156**

**Table 3:** Quantitative comparison of the Pareto fronts on the IEEE 68-bus system.

Method	RR_99_ (%)	RR_100_ (%)	HV
Single CNN	13.69%	20.87%	9.79
ConfidNN	11.88%	17.25%	13.07
Multi-kernel CNN ensemble	11.06%	15.31%	14.86
BNN	13.16%	19.63%	10.73
CNN-based bootstrap ensemble	11.50%	15.19%	14.16
CNN-based deep ensemble	**10.** **50** **%**	**13.62%**	**16.08**

**Table 4:** Prediction performance with typical thresholds on the IEEE 68-bus system.

Case	Class-Specific Thresholds		Validation Set			Test Set		
	*T_u_*	*T_s_*	TSR (%)	TUR (%)	RR (%)	TSR (%)	TUR (%)	RR (%)
1	0.698	0.969	99.06	100	10.50	99.34	100	11.00
2	0.759	0.969	99.43	100	11.56	99.53	100	11.50
3	0.811	0.969	99.72	100	12.12	99.71	100	11.88
4	0.866	0.969	99.90	100	13.25	99.90	100	12.75
5	0.895	0.969	100	100	13.62	100	100	13.12

**Table 5:** DO comparison on the NPCC 140-bus system.

Method	DO
Single CNN	0.306
ConfidNN	0.270
Multi-kernel CNN ensemble	0.243
BNN	0.279
CNN-based bootstrap ensemble	0.230
CNN-based deep ensemble	**0.140**

**Table 6:** Quantitative comparison of the Pareto fronts on the NPCC 140-bus system.

Method	RR_99_ (%)	RR_100_ (%)	HV
Single CNN	14.53%	19.97%	5.76
ConfidNN	10.77%	14.27%	9.40
Multi-kernel CNN ensemble	9.40%	14.63%	9.58
BNN	10.67%	16.27%	10.85
CNN-based bootstrap ensemble	9.23%	12.30%	11.39
CNN-based deep ensemble	7.70%	11.00%	13.03

**Table 7:** Prediction performance with typical thresholds on the NPCC 140-bus system.

Case	Class-Specific Thresholds		Validation Set			Test Set		
	*T_u_*	*T_s_*	TSR (%)	TUR (%)	RR (%)	TSR (%)	TUR (%)	RR (%)
1	0.574	0.995	99.06	100	7.70	99.25	100	9.10
2	0.640	0.995	99.42	100	8.37	99.41	100	9.60
3	0.720	0.995	99.63	100	8.97	99.57	100	9.93
4	0.851	0.995	99.89	100	10.37	99.89	100	11.60
5	0.899	0.995	100	100	11.00	100	100	12.43

**Table 8:** Computational costs analysis of the single CNN and the ensemble model.

System	M	Single CNN Training Time	Ensemble Model Training Time	Ensemble Model Inference Time
IEEE 68-bus system	10	1.30 min	13.02 min	0.43 ms
NPCC 140-bus system	20	3.78 min	75.60 min	0.96 ms

## Data Availability

The source code of this work is available at https://github.com/Treeyew671/Reliable-TSP (accessed on 20 July 2026).
